# Watered-Based Graphene Dispersion Stabilized by a Graft Co-Polymer for Electrically Conductive Screen Printing

**DOI:** 10.3390/polym15020356

**Published:** 2023-01-10

**Authors:** Fengfeng Zhao, Hui Quan, Shijun Zhang, Yihui Xu, Zheng Zhou, Guangxin Chen, Qifang Li

**Affiliations:** 1 College of Material Science and Engineering, Beijing University of Chemical Technology, Beijing 100029, China; 2 Sinopec (Beijing) Research Institute of Chemical Industry Co., Ltd., Beijing 100013, China; 3 Sinopec Group, Beijing 100728, China

**Keywords:** graphene, conductive inks, graft copolymer, aqueous solution

## Abstract

Graphene conductive inks have attracted significant attention in recent years due to their high conductivity, corrosion resistance, and environmentally friendly nature. However, the dispersion of graphene in aqueous solution is still challenging. In this work, we synthesized an amphiphilic graft copolymer, polyvinyl alcohol-g-polyaniline (PVA-g-PANI), and studied the graphene dispersion prepared with the graft copolymer by high-speed shear dispersion. The amphiphilic graft copolymer can be used as a stabilizer and adhesive agent in graphene dispersion. Given the steric hindrance of the graft copolymer, the stability of graphene dispersion is improved by decreasing the probability of π–π stacking. PVA-g-PANI has a better stability on graphene dispersion than carboxymethylcellulose sodium (CMC-Na) and a mixture of PVA and PANI. The graft copolymer has only a slight effect on the conductivity of graphene dispersion due to the existence of conductive PANI, which is beneficial for preparing the graphene dispersion with good conductivity and adhesion. Graphene dispersion is well-adapted to screen printing and is very stable with regard to the sheet resistance bending cycle.

## 1. Introduction

With the rapid development of the electronic industry, conductive ink has been applied in various fields, such as flexible printing circuits, film switcsh, and radio frequency identification [1,2]. Graphene, as the basic building block of other carbon materials, consists of a monolayer of graphitic carbon with sp2-bonded carbon atoms, whose unique structure gives it many excellent properties, such as tensile strength, Young’s Modulus, electrical conductivity, thermal conductivity, specific surface area, and high barrier properties [3,4,5,6]. Using graphene as a filler in conductive ink has the advantage of having excellent conductivity, corrosion resistance, and oxidation resistance [7,8]. Therefore, graphene conductive ink has attracted significant attention in recent years and has been one of the most widely used printed electronic materials.

Graphene in water can easily agglomerate and hardly obtains a stable aqueous solution due to its unique surface energy [9]. The agglomerated graphene cannot be formed at the macroscale, effective conductive network or superimposed on each other to form a dense film structure, limiting the application of graphene conductive ink. During the preparation of a highly conductive water-based graphene ink, if we can obtain highly stable graphene dispersion in aqueous solution, then we may be confident that graphene will have its own high conductivity. Therefore, stable dispersion is one of the most important prerequisites for the widespread application of graphene in composites and other materials. The methods of graphene dispersion mainly include physical and chemical dispersion [10]. Physical dispersion is achieved by adding surfactants to the solvent and using hydrogen bonding, π–π interaction, and electrostatic repulsion to maintain a stable dispersion of graphene [11,12,13]. Meanwhile, chemical dispersion is achieved by modifying graphene, but it leads to defects in the graphene sheets [14]. Many types of surfactants are typically divided into non-ionic, anionic, and cationic surfactants according to the difference in the hydrophilic part [15]. The adsorption of the different types of surfactants on the surface of graphene is different due to their different structures [16]. Regardless of the type of surfactant used, no obvious effect can be observed on the morphology and sheet size of graphene [17]. Bourlinos et al. [18] obtained a stable graphene aqueous solution by using polyvinylpyrrolidone as an assistant to exfoliate graphite. Ramalingam et al. [19] made stable graphene aqueous solution (2.58 mg/mL) with few layers by using sodium deoxycholate to assist exfoliate graphite. Narayan et al. [20] used pyrene-3,4,9,10-potassium tetracarboxylate to assist exfoliate graphite in liquid-phase exfoliation, which successfully produced 0.5–0.8 mg/mL of graphene dispersion. 

The synthesis and self assembly of copolymers have been extensively studied [21,22,23]. Some scientists designed new molecular structures and synthesized new efficient, green, and low-cost copolymers to improve the efficiency of exfoliate graphite and the stability of graphene dispersion. Cui et al. [24] synthesized four random copolymers based on poly(vinyl imidazole), which were used to assist the exfoliation of graphene in the aqueous solution; the highest concentration of 1.12 mg/mL graphene dispersion was prepared. Perumal et al. [25] designed and synthesized poly(ethylene oxide)-block-poly(4-vinylpyridine), which was used for the stabilization of graphene nanoplatelets in alcohol and water. Graphene concentrations up to 1.7 and 1.8 mg/mL were obtained from graphite platelets and reduced graphene oxide dispersions, respectively. Shin et al. [26] successfully prepared water-based, highly concentrated, stable, and defect-free graphene dispersions by using new cationic pyrenes. However, these surfactants are non-conductive and are difficult to remove from the graphene film, which adversely affects the conductive properties of graphene conductive ink.

Polyaniline (PANI) is one of the earliest conductive polymer materials that has attracted much attention due to its low cost, easy synthesis, unique doping mechanism, high conductivity, and excellent redox property [27,28,29,30]. Accordingly, this material is highly appealing for combining graphene and PANI to create high-performance conductive ink. When PANI is applied in graphene conductive ink, graphene and PANI can form a more continuous conductive network. Furthermore, PANI can be used as a spacer to further separate adjacent sheets of graphene. Xu et al. [31] prepared graphene/PANI conductive ink by ultrasound. The surface square resistance of the ink film was 846 Ω/cm^2^, which was used as a film electrode in the super capacitor, and the device has a longer cycle life. However, PANI has a low solubility in solvents and poor film-forming properties, which adversely affects its application progress. Polyvinyl alcohol (PVA) is a water-soluble polymer and has good biocompatibility, good film-forming properties, and the strength and flexibility of the film after forming can basically meet the requirements of film applications [32,33].

In this study, we designed and synthesized an amphiphilic graft copolymer and used it as a stabilizer and adhesive to improve the stability of the graphene aqueous solution, the electrical conductivity of graphene dispersion, and the adhesion of graphene film on Biaxially Oriented Polypropylene (BOPP). A stable and highly conductive graphene dispersion was obtained via the high-speed shear method. We studied the stability, screen printing adaptability, and physicochemical and electrical properties of the graphene dispersion. The introduction of PVA-g-PANI into graphene dispersion provides a new, environmentally friendly approach to preparing stable and uniform graphene aqueous dispersions.

## 2. Materials and Methods

### 2.1. Materials

Graphene (GS-1) was purchased from Ningbo Moxitech Co., Ltd., Ningbo, China. Poly(vinyl alcohol) (PVA with hydrolysis of 88%, Mw ~24,000), ammonium persulphate (APS), and CMC-Na (Mw ~250,000) were obtained from Aladdin (Shanghai, China) and used without further purification. Epichlorohydrin (EPIC), dimethyl sulfoxide (DMSO), triethylamine, and acetone were purchased from J&K chemic Tech Co., Ltd., Tianjin, China. Aniline was purchased from Macklin (Shanghai, China) and purified before use. Tetrabutyl ammonium chloride (TBACl, 98%) was purchased from Tianjin Biochemical Tech Co., Ltd., Tianjin, China. NaOH and hydrochloric acid solution were purchased from Sinopharm Chemical Reagent Co., Ltd. (Shanghai, China). BOPP, polyimide, and polyethylene terephthalate film were obtained from the Beijing research institute of the chemical industry. 

### 2.2. Synthesis of Graft Copolymer

**Scheme 1:**
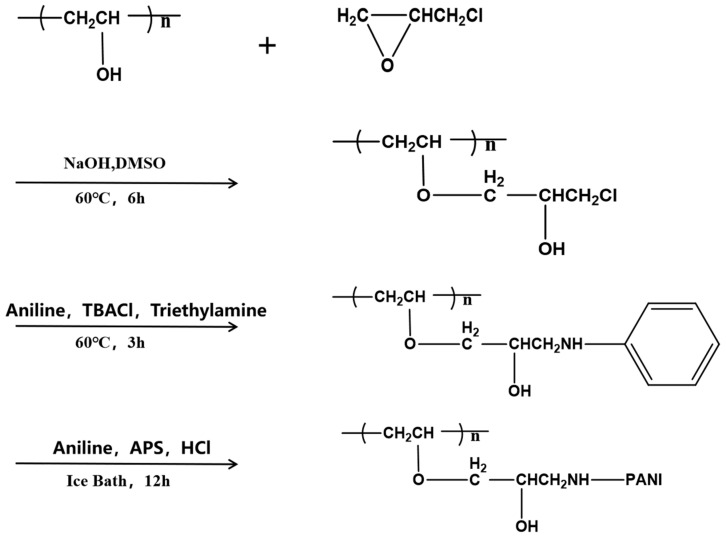
Synthesis of PVA-g-PANI.

Scheme 1 illustrates the synthesis of PVA-g-PANI. First, 15 g of PVA was dissolved in DMSO at 80 °C to obtain a solution. Approximately 1 g of EPIC was added into the solution, and the mixture was stirred at 30 °C for 30 min. Thereafter, 1 mL of 2.5 mol/L NaOH aqueous solution was added into the mixture and stirred at 60 °C. After 6 h, the mixture was filtered, and the precipitate was rinsed with acetone to obtain etherified PVA. Then, the etherified PVA, aniline, tetrabutylammonium chloride, and triethylamine were mixed with a molar ratio of 20:20:1:40 at 60 °C for 3 h, and the mixture was filtered to obtain a precipitate. Subsequently, the precipitate and 10 g of aniline were added into the 1 mol/L hydrochloric acid aqueous solution and stirred in an ice bath. The APS of the same molar ratio as aniline was slowly added dropwise over a period of 1 h. The experiment was performed for 12 h in an ice bath with constant stirring. Finally, the reaction mixture was filtered, and the filtrate was concentrated under reduced pressure. PVA-g-PANI was obtained by drying the concentrated filtrate in a vacuum at 30 °C for 48 h. 

### 2.3. Preparation of Graphene Dispersion Using Graft Copolymer

A stable dispersed graphene solution was prepared using the high-speed shear method. Firstly, 2.8 g of graft copolymer was directly dissolved in 105 mL of deionized water to obtain a solution. Then, 3.75 g of graphene was added into the solution, and the mixture was sheared by a high-speed shear dispersing emulsifier (TYPE R, LBX) to obtain a graphene dispersion. The experiments were performed for 1 h with a speed of 4000 rpm at room temperature. Furthermore, the prepared dispersions were deposited on substrates by using screen printing.

### 2.4. Characterization

FTIR spectra were recorded on a Tensor-27 spectrometer (Bruker, Karlsruhe, Germany). 1H NMR spectra were obtained on a Bruker AV400 spectrometer using deuterium oxide (D_2_O) as a solvent. The electrical properties of the films were measured using a four-probe tester (RTS-9, Zhejiang, China) and a multimeter (VC830L, Beijing, China). UV−Vis spectra measurements were carried out on a UV-1800 spectrophotometer, Karlsruhe, Germany. The adhesion test was measured using an adhesion tester (QFH) via the cross-cut test. The viscosity of the graphene dispersion was measured using a digital rotary viscometer (NDJ-5S, Jiangsu, China). TEM measurements were carried out on a transmission electron microscope (Tecnai G220, Waltham, MA, USA). SEM measurements were carried out on a scanning electron microscope (JCM-7000 NeoScope, Tokyo, Japan). Rheological characterization of the ink was conducted using a rotational rheometer (HAKKE MARS III, Waltham, MA, USA).

## 3. Results and Discussion

### 3.1. Synthesis of the PVA-g-PANI Graft Copolymer

**Figure 1:**
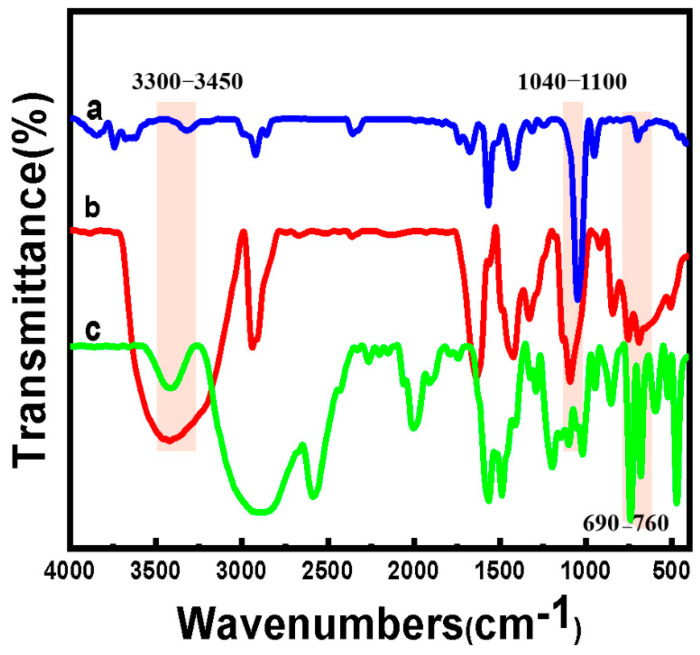
FTIR spectra of (**a**) etherified PVA, (**b**) N-alkylated product, and (**c**) PVA-g-PANI.

We developed a simple method to prepare PVA-g-PANI, as displayed in Scheme 1. Etherified PVA was obtained by using EPIC as an etherifying agent and connected with aniline via N-alkylation action to obtain an N-alkylated product. PVA-g-PANI was synthesized by the oxidative polymerization of the N-alkylated product and aniline in the aqueous acid (1 M HCl). Figure 1 presents the FTIR spectrum of the etherified PVA, N-alkylated product, and PVA-g-PANI. An –OH characteristic band was observed at 3300–3450 cm^−1^. The FTIR spectrum of the etherified PVA (Figure 1a) shows the C–O–C characteristic band at 1046 cm^−1^ and the C–Cl characteristic band at 712 cm^−1^, indicating that the etherified PVA was successfully prepared. The FTIR spectrum of the N-alkylated product (Figure 1b) shows the C–O–C characteristic band at 1094 cm^−1^ and the characteristic band of aniline at 693 and 759 cm^−1^. Moreover, the C–Cl characteristic band disappeared, indicating that the etherified PVA and aniline were successfully connected. The PVA-g-PANI (Figure 1c) presents the characteristic bands of PVA and PANI compared with the etherified PVA (Figure 1a). 

**Figure 2:**
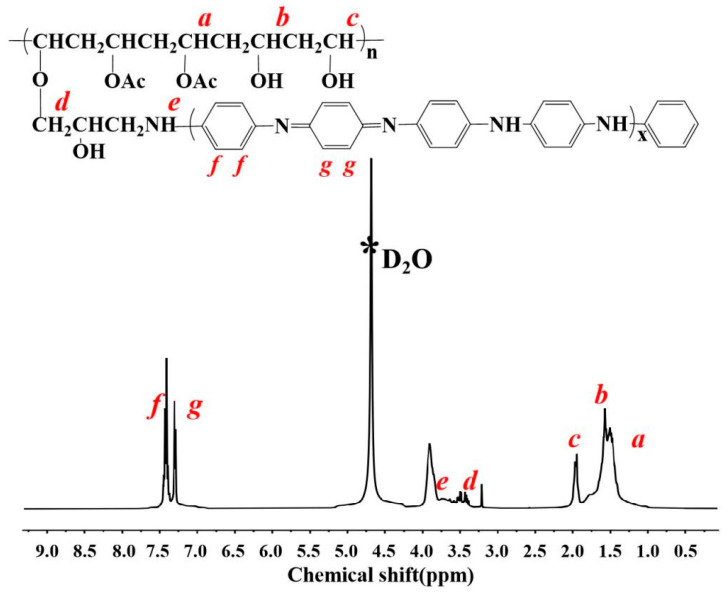
1H NMR of graft copolymer PVA-g-PANI.

Figure 2 presents the 1H NMR spectrum of PVA-g-PANI. Three proton signals (a, b, and c) of the methylene group of PVA and the proton signals (e) of –OH of PVA were observed at 1.40–2.00 and 3.88 ppm. The proton signals (d) of the methylene group at 3.53 ppm were also observed, demonstrating that the etherified PVA was successfully obtained. Meanwhile, the proton signals of tertiary amine at 3.76 ppm can be clearly observed, indicating that the etherified PVA was successfully connected with aniline via N-alkylation action. The aromatic protons (f and g) of PANI at 7.25–7.50 ppm were also observed. The 1H NMR spectrum confirmed that PVA-g-PANI was successfully synthesized.

### 3.2. Stability of Graphene Dispersion Prepared with a Graft Copolymer

**Figure 3:**
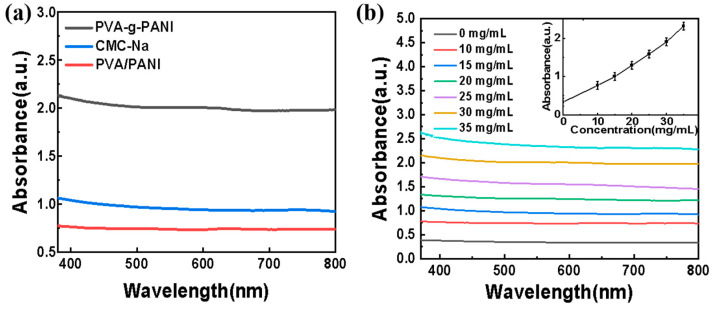
UV–Vis absorption spectra of graphene dispersion prepared with (**a**) different surfactants and (**b**) varying concentrates of PVA-g-PANI.

Graphene dispersion prepared with PVA-g-PANI is a viscous paste with a viscosity of 8000 mpa·s (Table 1), which is suitable for screen-printing technology. The concentration of PVA-g-PANI has no effect on the viscosity of the graphene dispersion, and thus it is not disadvantageous for the screen printing adaptability of graphene dispersion. The UV−Vis absorption spectrum was used to estimate the stability of graphene dispersion. The absorption intensity follows Beer’s law, and the higher the absorbance, the higher the concentration of graphene. PVA-g-PANI can significantly enhance the stability of graphene dispersion compared with CMC-Na, a common surfactant for graphene ink, and the mixture of PVA and PANI (Figure 3a). A strong π–π conjugation can be observed between the aromatic structure of PANI and graphene sheets, allowing PVA-g-PANI to be firmly attached to the surface of the graphene sheets. Meanwhile, the structure of PVA-g-PANI is a comb structure, which can produce a stronger steric hindrance (Figure S1). Theoretically, PVA-g-PANI can effectively prevent graphene sheets from agglomerating in the aqueous solution. The absorbance of graphene dispersion is higher when a high concentration of PVA-g-PANI was used, and it is greater than the absorbance of graphene dispersion without PVA-g-PANI, demonstrating that PVA-g-PANI can effectively improve the stability of graphene in the aqueous solution (Figure 3b). The critical micelle concentration of the PVA-g-PANI solutions is about 8 mmol/L (Figure S2).

**Figure 4:**
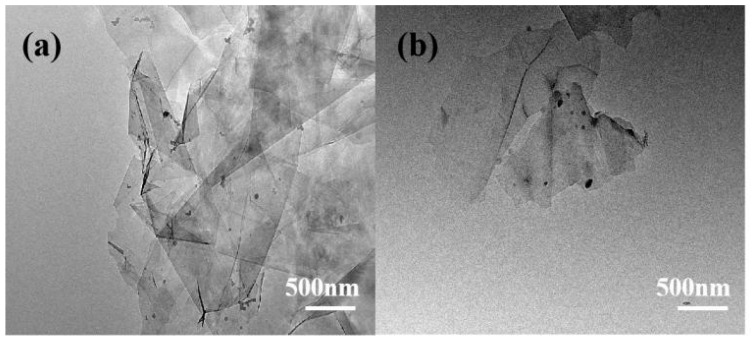
TEM images of (**a**) graphene dispersion and (**b**) graphene dispersion prepared with PVA-g-PANI.

The TEM images of graphene dispersion and graphene dispersion prepared with PVA-g-PANI are shown in Figure 4. Significant agglomeration and stacked sheets can be observed in graphene dispersion prepared with nothing (Figure 4a). When PVA-g-PANI is used to stabilize the graphene dispersion, most graphene sheets in the dispersion are large sheets with few layers (Figure 4b), confirming that PVA-g-PANI is effective in preventing the agglomeration of graphene. Raman data were shown in Figure S3, which indicates that the graphene was not modified by the copolymer.

### 3.3. Graphene Ink Prepared with a Graft Copolymer for Screen Printing

**Figure 5:**
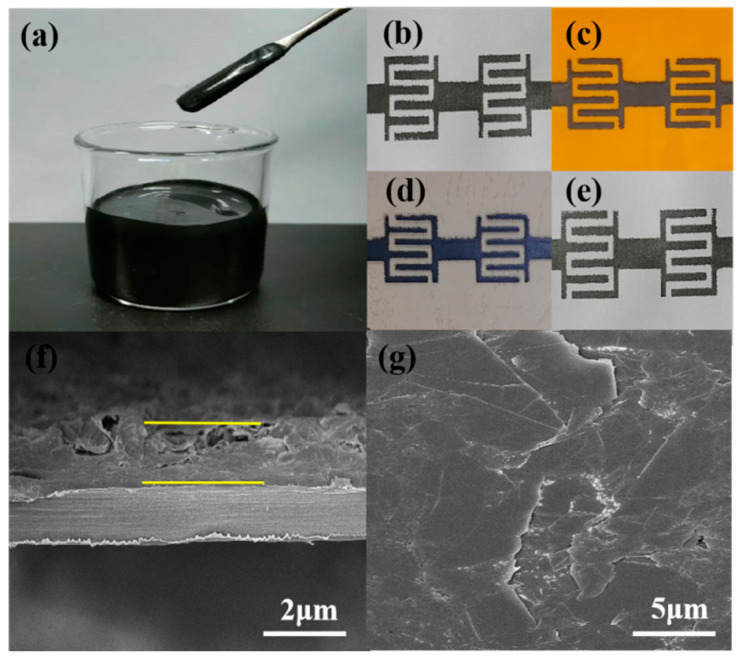
(**a**) Graphene/PVA-g-PANI ink; graphene/PVA-g-PANI ink at different substrates: (**b**) BOPP, (**c**) PI, (**d**) PET, and (**e**) A4 paper; SEM images of the film prepared with graphene/PVA-g-PANI ink: (**f**) section and (**g**) surface images.

The graphene ink prepared with PVA-g-PANI is shown in Figure 5a, and it presents as a viscous paste with high static viscosity. This ink can be printed on a widely used BOPP film by using a screen-printing technology and on PI, PET, and A4 paper substrates (Figure 5b–e). Furthermore, as shown in Figure S4, the surface of patterns prepared by graphene dispersion is flat and smooth. The morphology of the graphene ink prepared with a graft copolymer is shown in Figure 5f,g. The graphene sheets closely overlap, and the gap between the sheets is minimal, forming a dense graphene film and a complete conductive network, which is beneficial for the conductivity of the graphene ink.

**Figure 6:**
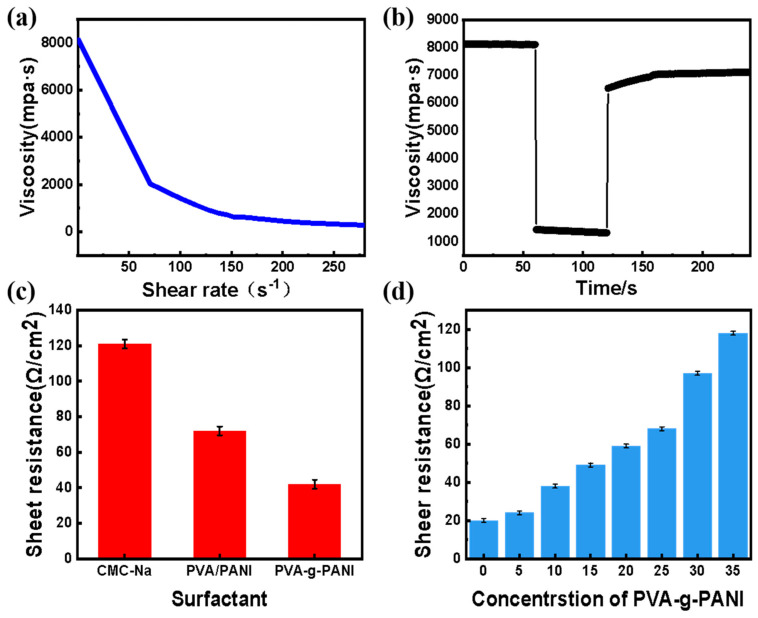
(**a**) Viscosity as a function of shear rate for the graphene/PVA-g-PANI ink, (**b**) graphene/PVA-g-PANI ink rheological behavior during simulation of screen printing, (**c**) sheet resistance of graphene ink with different surfactants, and (**d**) sheet resistance of graphene ink with different concentrations of PVA-g-PANI.

When the ink has appropriate rheological characteristics, we can obtain the printing pattern that has more smooth edges and a higher resolution through screen-printing technology. Furthermore, the defects and bubbles in the printing patterns must be eliminated, and the conductivity of the printing patterns must be improved. In Figure 6a, the viscosity of the graphene dispersion decreases with the increase in the shear rate, indicating that graphene dispersion has a significant shear-thinning behavior, which is beneficial for the extrusion of graphene dispersion in the printing process. The modulus recovery of graphene dispersion studied by three interval tests is shown in Figure 6b. First, graphene dispersion was applied at a low-stress oscillatory (10 Pa for 60 s), then at a high-stress oscillatory (100 Pa for 60 s), and, finally, at a low-stress oscillatory (10 Pa for 120 s). The storage modulus of the ink can be quickly restored after 100 pa shear stress, indicating that the destroyed network of the graphene dispersion can be quickly rebuilt, and a higher resolution of printing patterns can be obtained.

In Figure 6c, the graphene ink prepared with PVA-g-PANI has a lower sheet resistance compared with CMC-Na and the mixture of PVA and PANI, indicating that PVA-g-PANI is beneficial in forming a more complete conductive network. Meanwhile, the sheet resistance of the printing patterns slightly increases as its concentration increases when PVA-g-PANI was used (Figure 6d). This phenomenon occurred because PVA-g-PANI contains non-conductive PVA and is difficult to remove from the graphene film, which adversely affects the conductive properties of the printing patterns of graphene dispersion.

**Figure 7:**
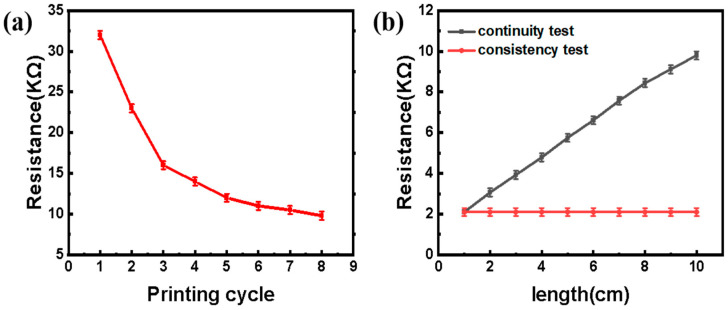
(**a**) Resistance of the graphene/PVA-g-PANI ink at different printing cycles; (**b**) resistance consistency and continuity of the graphene /PVA-g-PANI ink.

The screen printing pattern was cut into 4 mm wide and 10 cm long straight lines. The resistance of the line gradually decreases with the increase in printing cycles. When the printing time is up to eight, the resistance can decrease from the initial 32 KΩ to 9.8 KΩ (Figure 7a). This phenomenon occurs because multiple printing can increase the filling amount of graphene in the line segment. When graphene reaches a certain critical content, the conductive network is complete. Accordingly, the decrease in resistance is not that obvious anymore. The Figure 7b demonstrates that the resistance of the corresponding line increases linearly, indicating that the printing line segment has no fault and that it has continuity. The 10 cm line was divided into 10 segments on average, and the resistance of each segment was measured. The resistance of each segment is about 2.1 KΩ, as shown in the red dotted chart, showing that the resistance of the printing line has consistency. The results also show that the prepared PVA-g-PANI/graphene ink was uniformly dispersed to a certain extent.

**Figure 8:**
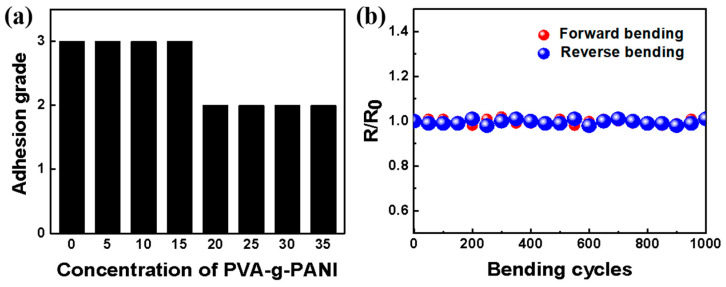
(**a**) Adhesion grade of the graphene/PVA-g-PANI ink on the BOPP substrate; (**b**) bending stability of the graphene/PVA-g-PANI film.

The cross-cut test was used to estimate the adhesion of the graphene/PVA-g-PANI film on the flexible BOPP substrate. The result is shown in Figure 8a. When the concentration of PVA-g-PANI is over 15 mg/mL, the adhesion grade is up to 2, indicating that the damage to the graphene/PVA-g-PANI film is less than 15% after the cross-cut test, and the adhesion of the graphene/PVA-g-PANI film meets the requirements for general use. This situation occurs because PVA-g-PANI contains PVA with good film-forming and wetting effects for substrates, which are beneficial for forming a dense and flexible graphene film and improving the adhesion to substrates. During 1000 bends, the change in sheet resistance (R/R0) of the graphene/PVA-g-PANI film slightly fluctuates in the forward bending and reverse bending (Figure 8b). Accordingly, the sheet resistance of the printed pattern is not affected by multiple bends and folds. Thus, the graphene/PVA-g-PANI ink is very stable regarding sheet resistance bending cycles, making it potentially useful in a variety of applications.

## 4. Conclusions

We presented a study on the stability, screen printing adaptability, and electrical properties of graphene dispersions using amphiphilic graft copolymers of PVA-g-PANI. The stable graphene dispersion was prepared via high-speed shear. 1H NMR and FTIR were used to characterize the structure of graft copolymers, and their results confirm that amphiphilic PVA-g-PANI was successfully synthesized. The UV–Vis absorption spectral results and the TEM images confirm that PVA-g-PANI can efficiently improve the stability of graphene in the aqueous solution, better than CMC-Na and the mixture of PVA and PANI. Moreover, the PVA-g-PANI is beneficial for preparing the graphene dispersion with good conductivity. The prepared graphene dispersion is very adaptable for screen printing and is very stable regarding the sheet resistance bending cycle. This study offers a simple, scalable, and environmentally friendly approach to produce stable and uniform graphene aqueous dispersions via a high-speed shear dispersing method. 

## Figures and Tables

**Table 1:** Effect of different concentrates of PVA-g-PANI on graphene dispersion.

Concentrates of PVA-g-PANI (mg/mL)	0	10	15	20	25	30	35
Viscosity (×10^3^ mpa·s)	8	8	8	8	8	8	8

## Data Availability

The data presented in this study are available on request from the corresponding author.

## Supplementary Materials

The following are available online at https://www.mdpi.com/article/10.3390/polym15020356/s1, Figure S1: Schematic representation of the stabilization mechanism; Figure S2: Conductivity of the micellar solutions with different concentration; Figure S3: Raman spectra of graphene and graphene with PVA-g-PAN; Figure S4: The patterns of graphene dispersion on (a) A4 paper; (b) PET.
