# Supplementary material for: Watered-Based Graphene Dispersion Stabilized by a Graft Co-Polymer for Electrically Conductive Screen Printing
Source: Polymers (Basel). 2023 Jan 10;15(2):356. doi: 10.3390/polym15020356 (PMC9860939; doi:10.3390/polym15020356)
Supplement: Supplementary file 1 [file polymers-15-00356-s001.zip › polymers-2006492-supplementary.pdf]

Watered-based graphene dispersion stabilized by a graft co-polymer for electrically conductive screen printing

Supporting information

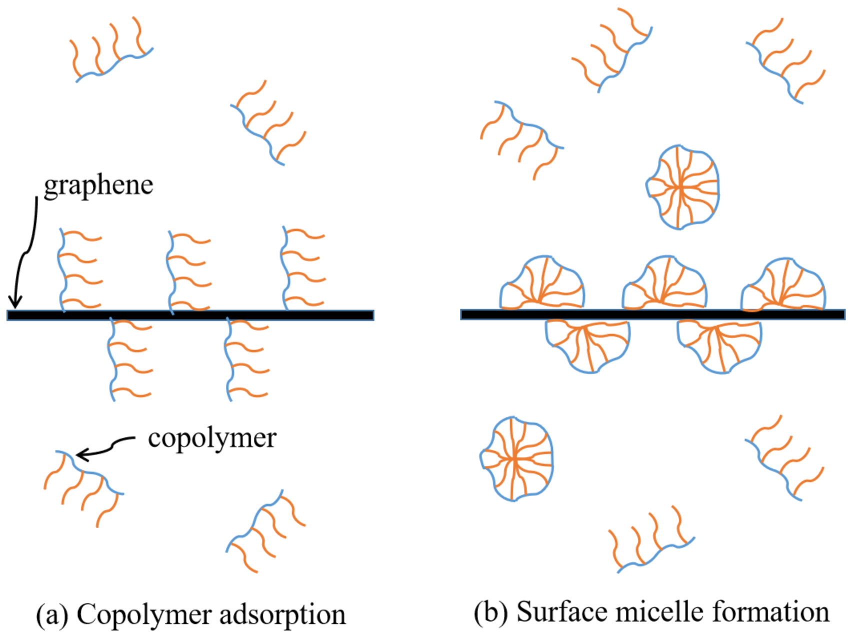

Figure S1. Schematic representation of the stabilization mechanism

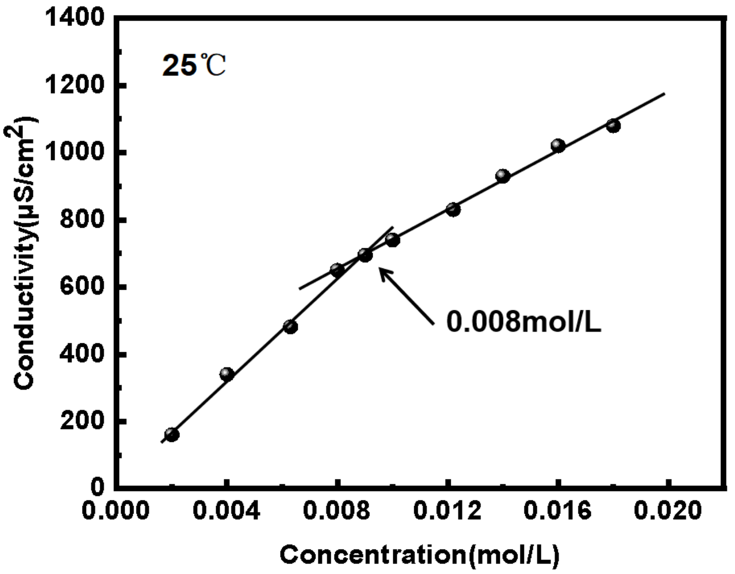

Figure S2. Conductivity of the micellar solutions with different concentration

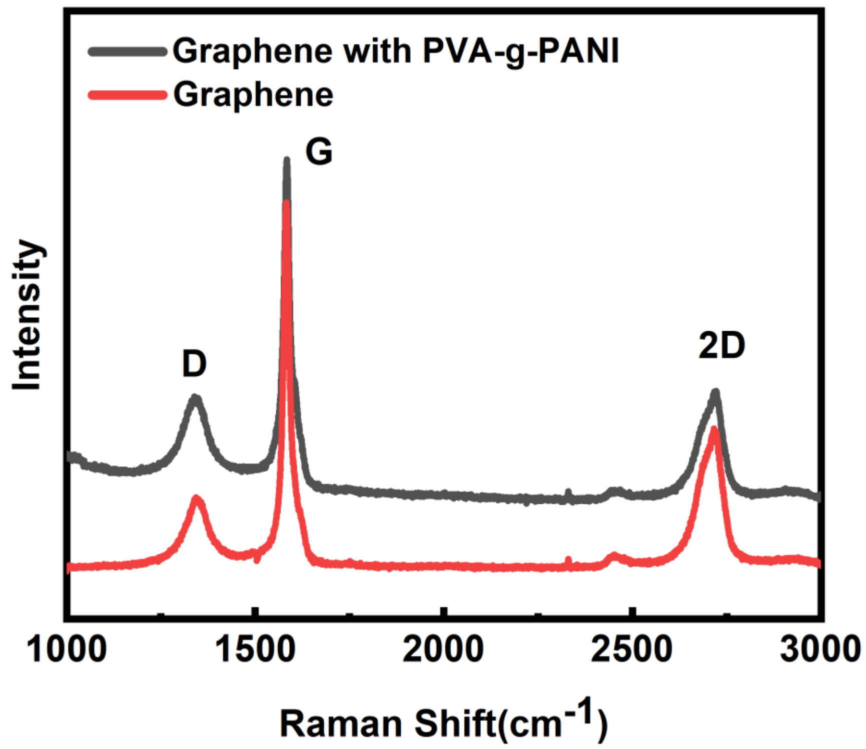

Figure S3. Raman spectra of graphene and graphene with PVA-g-PAN

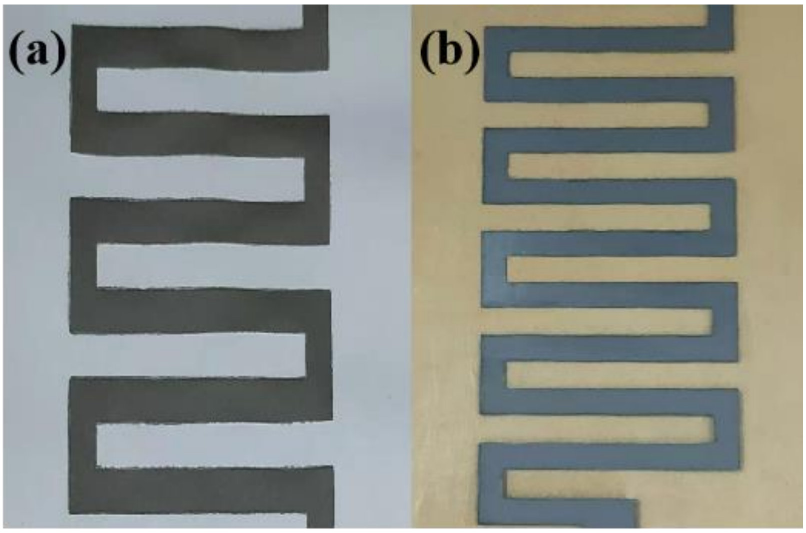

Figure S4. The patterns of graphene dispersion on (a)A4 paper; (b) PET.
